# Modern Sociology

**Published:** 1898-10-22

**Authors:** 


### Modern Sociology.

### PEOPLE'S BANKS.

### IV.—What About England?

People's banks, similar to those we have described, have been established in Switzerland, France, Belgium, and Austria-Hungary, and everywhere seem to have done good work in lifting the small tradesman or peasant farmer out of the whirlpool of usury into which he is apt to fall; but as these are in all essentials the same as their parents, it is unnecessary to deal with them in detail. The question for us is, Can these institutions be acclimatised here? On the face of it the answer would be "No." We have not here the peasant proprietor, as on the Continent, nor to so great an extent the small jobbing artisan, who does a little of everything, to whom the people's banks are such a boon abroad. On this side of the Channel the tendency has been towards the agglomeration of business into large concerns, leaving only the smallest scope for individual energy in our lesser towns and villages. In one sense this is to be regretted, if it gives the average man the feeling that he can never be aught but a man under authority—a man bound to obey without inquiring "why" the orders of an intelligence beyond his own. But even accepting this as inevitable, would not banking facilities greater than he now possesses be useful to the shopkeeper in small country towns and villages?

We have, indeed, savings banks—the post office one, and some few private ones. We are very far from ignoring the good work done by these in encouraging thrift, although the small interest they give, and the restrictions under which they work forbid their being anything more than the temporary resting-place of the savings of the industrious poor. But a bank must do more than accept deposits, if it is to be thoroughly useful to a community. It is a slow business accumulating enough in a savings bank to start in business on one's own account. How much more inviting would it be if savings to a certain amount, with implied proof given by these of probity, thrift, and energy, could be rewarded by an advance of money at a reasonable rate of interest, to enable one to set up in business, to purchase a house or a plot of land, and so improve one's position in the world? Saving for saving's sake has attractions for but few; most of us hope to find our economy rewarded by greater comfort and freedom from care in our old age, or for our children after us. Thus many would save, if they saw in such savings as are possible for them the chance of rising to greater affluence. Two and a half per cent. may be sufficient attraction to a man who has a considerable amount of capital, to save still more, but it is not enough for a man who sees no chance of saving more than £100 or £200 in his lifetime. He requires something better.

Now, the people's bank, which meets saving with a loan of money, to be spent on some specific reproductive object, is in itself a great incentive to thrift. If a man cherishes an ambition to buy a plot of land, or set up in business of some sort, it is a great encouragement to him to know that if he can show as much thrift, self-control, and industry as will enable him to save part of the purchase-money, a bank will advance him enough at a fair rate to complete the purchase, or make such improvements as may be desirable. Saving now begins to have a meaning, a purpose which before was wanting to it. Or being in business, in a small way, he may see some opportunity of advancing himself, if only he had a little ready money. The man in a small way of business very rarely thinks of going to an ordinary bank. Either he lets the golden opportunity slip, or he goes to some usurer, whose interest eats up all the profit, even if it does not bring the unfortunate adventurer to utter ruin. The recent revelations of the usury committee show how many people go to a man who advances money without much preliminary inquiry, and charges an enormous interest, when they might reasonably get accommodation from a bank at a moderate rate. They pay a hundred per cent., when, at the outside, ten might do. And they could pay ten and prosper, while the hundred ruins them. But it does not occur to them that the bank in their town is an institution to apply to in their need. Bigger men—with bigger debts, perhaps—go there, and find help; but the man in a small way of business has never been accustomed to look to the bank for accommodation. In Scotland, through the cash-credit system, tradesmen in a very moderate business are more in the habit of dealing with a bank than they are in England; but even in Scotland, the small affairs of the people's bank—the loans of five or ten pounds for a year, on personal security, are almost unknown. There is therefore a class of people who, when they are in temporary need, fly either to the pawnshop or to the usurer, and we all know how that ends.

There are, indeed, many bankruptcies which are truly tragic; cases in which a man has bought a large stock, and has not been able to realise it in time to pay the wholesale houses which supplied it. With capital to keep him going for another year or so he could get on; but without that or its equivalent in credit, this is impossible. If the establishment of a people's bank would help to prevent these failures, to encourage people who are really honourable and industrious, they would be well worth having.

But, be it understood, they must be established by the people themselves. Government money so applied would in all probability be wasted. Napoleon III. gave a great deal to set up such loan institutions in France. The money was lent, indeed, but it was never repaid, and the result was not to encourage thrift and industry, but the reverse. A bank administered by managers who have no responsibility in connection with it—above all, who think they have the bottomless purse of the nation to dip into—is foredoomed to economic failure; the only safeguard lies in those who lend the money lending their own, and in those who borrow, borrowing what is in part their own. It is important, too, that a loan, whether for a long or short period, should be for some specifically stated purpose. This implies that it should be possible for the bank authorities, whoever they are, to exercise some supervision over their borrower's actions; and this implies, in turn, that the bank should be so limited in scope that such supervision would be practicable and easy. The village or the parish, as in the Raffeisen plan, would be large enough. To what extent the plan would be even then adapted to English circumstances it is not easy to say without making the experiment. But the experiment would be worth trying.

